# The impact of national non-pharmaceutical interventions (‘lockdowns’) on the presentation of cancer patients

**DOI:** 10.3332/ecancer.2021.1180

**Published:** 2021-02-03

**Authors:** Arnie Purushotham, Graham Roberts, Kate Haire, Joanna Dodkins, Elizabeth Harvey-Jones, Lu Han, Anne Rigg, Claire Twinn, Conjeevaram Pramesh, Priya Ranganathan, Richard Sullivan, Ajay Aggarwal

**Affiliations:** 1 King’s College London, WC2R 2LS, UK; 2 Guy’s and St Thomas’ NHS Foundation Trust, London, SE1 9RT, UK; 3 London School of Hygiene and Tropical Medicine, WC1E7 HT, UK; 4 Tata Memorial Cancer Centre, Homi Bhabha National Institute, Mumbai 400012, India

**Keywords:** COVID-19, pandemic, cancer, diagnosis, stage, shift

## Abstract

One of the most ignored aspects of the COVID-19 pandemic has been the impact of public health measures by governments on wider health and welfare. From March 2020, hospitals in the UK saw a dramatic reduction in patients with cancer presenting due to multifactorial reasons. The impact of the pandemic on patients with cancer in the South East London Cancer Alliance was studied. The specific aims were (1) to examine the reduction in cancer diagnoses during the first wave of the pandemic and (2) to examine the stage of diagnosis of patients with cancer presenting during the pandemic compared with that of patients presenting before the pandemic.

There was an 18.2% reduction in new cancer diagnoses (an estimate of 987 cancers), when compared with 2019. This fall in cancer diagnoses was most marked in patients with prostate (51.4%), gynaecological (29.7%), breast (29.5%) and lung (23.4%) cancers. There was an overall 3.9% increase in advanced stage presentation (Stages 3 and 4), with an overall 6.8% increase in Stage 4 cancers during this period. The greatest shifts were seen in lung (increase of 6.3%, with an 11.2% increase in Stage 4 cancer alone) and colorectal (5.4%) cancers. For prostate cancer, there was an increase in 3.8% in those presenting with Stage 4 disease. For breast cancer, there was an 8% reduction in patients diagnosed with Stage 1 cancer with commensurate increases in the proportion of those with Stage 2 disease.

The experiences in cancer are a salient warning that pandemic control measures and policy need to balance all health and welfare. Alternative strategies need to be adopted during further waves of the current and any future pandemic to ensure that patients with cancer are prioritised for diagnosis and treatment to prevent late-stage presentation and an increase in avoidable deaths.

## Background

One of the most ignored aspects of the COVID-19 pandemic has been the impact of public health measures—non-pharmaceutical interventions (NPI)—by governments on wider health and welfare. NPI measures such as ‘stay at home’ orders coupled with changes in public behaviour due to fear of hospitals and reduction in provision of services, e.g., surgery, has radically changed health ecosystems. Patients with new symptoms suggestive of cancer have chosen to delay their attendance with primary healthcare or hospital practitioners due to concern about attending healthcare facilities and the risk of contracting SARS-COV-2 [1] or because of compliance with government orders. Furthermore, the suspension of cancer screening and the dramatic reduction in endoscopic and cancer surgical services resulted in fewer patients with a new cancer diagnosis being referred and treated [2–6]. From March 2020, hospitals in the UK saw a dramatic reduction in cancer patients presenting through the urgent 2-week cancer pathway and cancer screening services. The reasons were multifactorial and included government ‘Stay at home’ public health messaging during the peak of the pandemic and cessation of breast, colorectal and cervical cancer screening services. In addition, there was significant contraction of diagnostic and treatment services, particularly endoscopy as the pandemic began to unfold.

As a result of healthcare systems reassigning resources in preparation for the COVID-19 pandemic, cancer surgery and radiotherapy have been deferred and the use of systemic treatments reduced [7, 8] despite calls to prioritise cancer patients’ treatment [9]. The direct impact of this strategy on patients with cancer is unclear. Modelling studies suggest that as a result of COVID-19, there will be in future, a significant impact on late-stage presentation and the number of avoidable cancer deaths [10, 11]. It has been predicted that for four major tumour types—breast, colorectal, lung and oesophago-gastric cancers, diagnostic delay alone during the pandemic will result in 3,500 additional cancer deaths due to the pandemic and up to 60,000 life years lost. However, to date, there has been no examination of actual trends in hospitals.

The South East London Cancer Alliance is a major cancer hospital network which encompasses three NHS trusts (Guy’s & St Thomas’ NHS Trust, King’s College Hospital NHS Trust and the Lewisham and Greenwich NHS Trust) covering a large (c. 2 million), socio-demographically diverse population. The population served by this network is representative of other large conurbations within the United Kingdom (National Cancer Registration and Analysis Service). This network treats all major cancers providing diagnostic and specialist treatment services with just under 8,000 cancer diagnoses each year.

In this study, we consider the impact of the COVID-19-pandemic NPI on patients with cancer in this network. Specifically, we aim to (1) to examine the reduction in cancer diagnoses during the first wave of the COVID-19 pandemic and (2) to examine the stage of diagnosis of patients with cancer presenting during the pandemic compared with the stage of diagnosis of cancer patients presenting before the pandemic across several tumour types. We hypothesise that as a result of the reduction of patients with cancer presenting to primary or secondary care, there will be a significant shift in stage at diagnosis with an increase in Stages 3 and 4 presentation across multiple tumour types. Stage migration was assessed in four ‘bell weather’ tumour types: breast, colorectal, lung and prostate cancer. These are the four commonest cancer types in the UK accounting for 55.6% of all cancers. They are also characterised by differences in age profile of patients; screening protocols and diagnostic investigations and provide an opportunity to understand better the differential impact of the COVID-19 pandemic on the cancer referral and diagnostic pathway.

## Methods

### Study population

- All patients newly diagnosed with cancer in the South East London Cancer Alliance that were discussed at a tumour-specific multidisciplinary meeting between January 2019 and September 2020 were included in the study. Any diagnoses marked as recurrence or progression were excluded. In all, a total of 5,423 new cancers were diagnosed during this period.
- A sub-study examined in more detail the fall and recovery trends of new diagnoses in patients with breast (International Statistical Classification of Diseases and Related Health Problems (ICD) C50), colorectal (ICD C17-C21), gynaecological (ICD C51-C58), head and neck (ICD C00-C14, C30-C32), lung (ICD C33-C37, C45) and prostate (C61) cancer in the South East London Cancer Alliance.
- A further sub-study focused on patients diagnosed with breast, colorectal, prostate and lung cancer at Guy’s & St Thomas’ NHS Trust to assess variation in stage of presentation in two 6-month periods before and after the introduction of pandemic lockdown measures. Staging data were collected between October 2019 and March 2020 and compared to stage at presentation between April 2020 and September 2020.

### Data sources

Data were sourced from four electronic cancer data collection systems across the South East London Cancer Network. This included (1) MOSAIQ electronic patient record (Guy’s and St Thomas’ NHS Trust), (2) Somerset Cancer Register (Guy’s and St Thomas NHS trust), (3) Somerset Cancer Register (King’s College Hospital), and (4) Somerset Cancer Register (Lewisham and Greenwich).

Staging information was missing in 10%–40% of patients through the electronic record system and therefore detailed case note review was used to supplement staging information to ensure close to 100% completion for the four tumour types in which stage migration was assessed.

### Statistical analysis

Chi squared test was undertaken to assess for any statistically significant differences in cancer stage at the time of cancer diagnosis pre and post first wave of the pandemic. All analyses were done using Stata version 16.

## Results

### Cancer diagnoses and recovery rates in South East London patients

**Figure 1.**
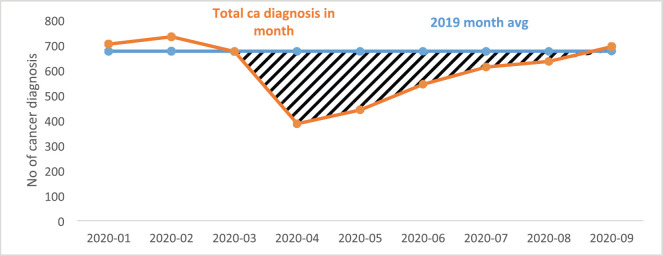
Total cancer diagnoses between January and September 2020 in South East London.

**Figure 2.**
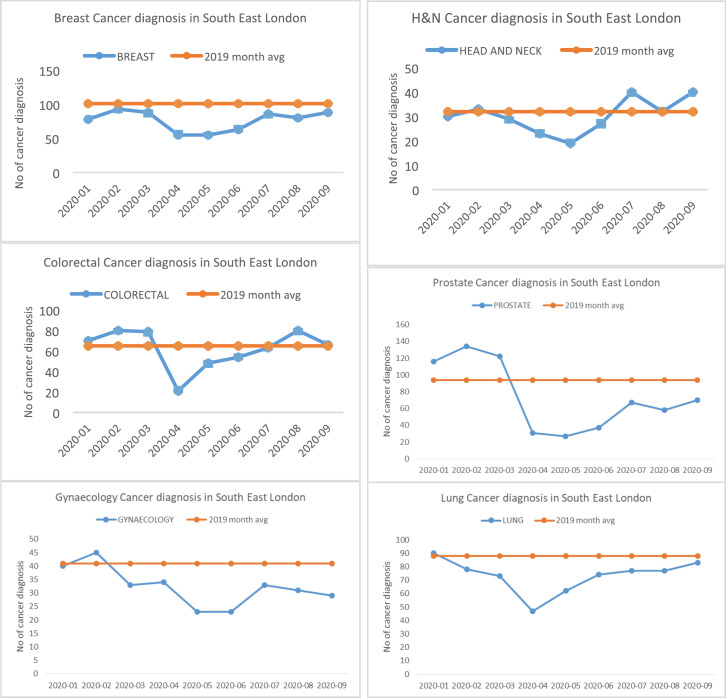
Fall in diagnosis and recovery trends in six tumour types—breast, colorectal, gynaecology, head and neck, prostate and lung.

In all, a total of 5,423 new cancers were diagnosed between 1 January and 30 September 2020. There was a significant fall in the number of newly diagnosed patients with cancer from March 2020 with a gradual recovery over subsequent months to expected levels by the end of September 2020 (Table 1; Figure 1). Overall, there was an 18.2% reduction in new cancer diagnoses (an estimate of 987 cancers), when compared with 2019 (mean monthly cancer diagnosis: 675 patients). This fall in the number of new cancer diagnoses was most marked in patients with prostate (51.4%), gynaecological (29.7%), breast (29.5%) and lung (23.4%) cancers. The fall in diagnoses and recovery trends in six tumour types: breast, colorectal, gynaecology, head and neck, prostate and lung is shown in Figure 2. Slow recovery rates were observed in breast, gynaecological, lung and prostate cancers (Figure 2).

### Stage of presentation at Guy’s and St Thomas

Cancer staging of patients presenting between April 2020 and September 2020 was compared with cancer staging of patients presenting between October 2019 and March 2020 (Tables 2 and 3). There was an overall 3.9% increase in advanced stage presentation (Stages 3 and 4), with an overall 6.8% increase in Stage 4 cancers during this period. The greatest shifts were seen in lung (absolute increase of 6.3%, with an 11.2% increase in Stage 4 cancer alone) and colorectal (5.4%) cancers (Tables 3 and 4). For prostate cancer, there was an absolute increase in 3.8% in those presenting with Stage 4 disease post pandemic. For breast cancer, there was an 8% reduction in patients diagnosed with Stage 1 cancer with commensurate increases in the proportion of those with Stage 2 disease.

Chi Squared Test was run to determine whether there was a statistically significant difference in the proportions of patients presenting with Stage 1, Stage 2, Stage 3 and Stage 4 disease post pandemic compared to the pre-pandemic cohort. This demonstrated no statistically significant difference in stage between the two cohorts for each of the cancer types.

## Discussion

The COVID-19 pandemic has had a significant negative impact on cancer diagnoses with fewer patients presenting during the first wave (UK lockdown in first wave from 20 March 2020), and an increase in the proportion of patients with breast, colorectal, lung and prostate cancer presenting with late-stage disease, which is likely to impact on their treatment and clinical outcomes.

Our results are in keeping with the findings of other studies. The UK modelling study [11] predicted an increase in avoidable deaths from breast, bowel and lung cancer due to delays in diagnosis during the pandemic, as a result of later stage at presentation and even at this early juncture in the pandemic our results suggest that stage migration is being observed. This will ultimately translate to an increase in deaths from cancer that otherwise could have been avoided. An observational study that compared patients presenting between January to April 2019 and January to April 2020 in 20 cancer institutions showed a reduction in cancer patients presenting with melanoma (52%), prostate (49%), breast (48%), colorectal (40%), lung (39%) and haemato-oncology (39%) cancers, predicting an increase in late-stage disease in future months [1]. The UK National Endoscopy Database analysed the impact of the pandemic on endoscopic services and cancer diagnosis during three periods, pre-COVID, transition and COVID-impacted [3]. This showed an average 82% reduction in endoscopic procedures. At its lowest point, this was 95%. Cancer detection rates fell by an average of 58% ranging from 19% in pancreatico-biliary cancer to 72% in colorectal cancer [3]. It is, therefore, essential that endoscopic capacity is rapidly increased to deal with the backlog of patients who will present with gastrointestinal symptoms suggestive of cancer. Furthermore, it is likely that patients who have not presented so far (i.e. missing) will probably present with very late stage disease.

Similarly, the breast and colorectal cancer screening programmes in the Netherlands were suspended to decrease pressure on health services overwhelmed by COVID-19 allow reallocation of staff and personal protective equipment (PPE) and to reduce the spread of COVID-19 [2]. As a consequence of this, there were fewer diagnosis of colorectal and breast cancer particularly in the screening age groups. This has gradually increased to expected levels, but it is unknown if as a consequence of this there will be stage migration which will alter clinical treatment and result in worse outcomes [2].

In order to prioritise the management of patients with cancer during the COVID-19 pandemic, clinicians have created treatment algorithms based on the likely impact of the delays in treatment on patients’ outcomes. For example, it is reasonable to defer treatment of low-grade non-muscle invasive bladder cancer and to prioritise muscle-invasive bladder cancer [6]. In addition, the creation of COVID-free pathways to manage patients may at least partially mitigate the potential increased risks of mortality.

Modelling studies have been conducted to determine the potential delay in cancer diagnosis on increased mortality [10, 11], it has been shown that modest delays of 3–6 months in cancer surgery will result in a significant impact on survival. These studies have examined the reduction in cancer referrals with potential outcome and have shown an increase in avoidable deaths. This is compounded even further by failure to increase diagnostic capacity to clear the backlog of cancer patients as referrals increase [10, 11].

A recent paper presented at the annual San Antonio Breast Cancer Conference in December 2020 showed that 64% fewer patients were diagnosed with breast cancer than a year ago and more patients presented with more advanced stage and aggressive sub-types of breast cancer during the COVID-19 pandemic compared with the same period in 2019 [12].

The South East London population contains some of the most deprived populations in England [13]. Our analysis was not able to determine, at this time point, whether socioeconomic status, geography, gender and/or age were associated with a statistically significant increase in stage migration.

Our findings have significant implications for economics of cancer care. It is well documented that late stage disease has higher direct healthcare costs [14], but furthermore the premature mortality and morbidity will also increase, indirectly, the economic burden on countries [15].

NPIs have also had an impact on patients’ comorbidities. At Guy’s and St Thomas NHS Trust, therapy and rehabilitation services have had to provide urgent/essential interventions with a focus around avoidance of admission and rapid interventions for symptom management to enable patients to continue treatment. Capacity to provide interventions to prevent deconditioning has been limited although virtual means have been utilised where possible to support patients and keep them well at home to avoid hospital admission. Patients presenting currently have lower levels of function, higher levels of symptom burden and require substantial input from therapy services to enable them to safely remain at home. Since coming out of lockdown and as face-to-face activity has increased, there has been a rapid rise in referrals for patients requiring urgent assessment. These patients are typically highly symptomatic and require rapid intervention from the therapies and rehabilitation teams to avoid admission, interruption in their cancer treatment and or delay in commencing treatment.

## Conclusion

These findings, although only from a part of the English NHS almost certainly reflect what is happening across the UK and elsewhere. In many countries, the impact of national lockdowns (NPI), fear of hospitals as sources of contagion and the reduction in the provision of cancer services are likely to have an even greater impact. Few countries have taken into account the indirect impact of public health measures on non-COVID-19 healthcare. The experiences in cancer are a salient warning that pandemic control measures and policy need to balance all health and welfare. Alternative strategies need to be adopted during further waves of the current pandemic and any future pandemics to ensure that patients with cancer are prioritised for diagnosis and treatment in order to prevent late-stage presentation and an increase in avoidable deaths. Analyses such as this should be updated regularly throughout the pandemic and beyond to understand diagnosis and stage shifts in real time to support global policy in the ongoing management of patients with cancer.

## Conflicts of interest

None.

## Funding source

None.

## Figures and Tables

**Table 1.** Total cancer diagnoses between January and September 2020 in South East London, broken down by individual tumour types.

TG	2020-01	2020-02	2020-03	2020-04	2020-05	2020-06	2020-07	2020-08	2020-09
Brain CNS	11	13	14	5	5	7	14	16	21
Breast	78	93	88	55	55	63	86	80	88
Colorectal	70	80	79	21	48	54	63	80	66
Gynaecology	40	45	33	34	23	23	33	31	29
Haematology	48	43	58	48	33	60	32	48	35
Head & Neck	30	33	29	23	19	27	40	32	40
HPB	30	24	21	28	31	21	23	39	45
Lung	85	74	70	46	61	71	72	73	77
Other	5	5	3	2	4	9	14	8	9
Prostate	108	131	115	28	25	36	65	53	67
Skin	100	93	95	50	80	89	98	103	130
Upper GI	39	51	33	19	30	43	26	34	44
Urology	60	48	36	27	27	41	46	38	43
**Overall**	**704**	**733**	**674**	**386**	**441**	**544**	**612**	**635**	**694**

**Table 2.** Cancer staging completeness for Guy’s and St Thomas patients (breast, colorectal, gynaecology, head and neck, lung and prostate).

Tumour group	Period	% staged
**Breast**	Oct 19 to Mar 20	100.0
	Apr 20 to Sep 20	99.4
**Colorectal**	Oct 19 to Mar 20	96.6
	Apr 20 to Sep 20	95.1
**Lung**	Oct 19 to Mar 20	99.2
	Apr 20 to Sep 20	96.2
**Prostate**	Oct 19 to Mar 20	98.8
	Apr 20 to Sep 20	93.6
**Overall**		97.9

**Table 3.** Cancer staging comparison Apr 2020 to Sep 2020 versus Oct 2019 to Mar 2020. Number of cases and percentage early versus late stage.

	Oct 2019 to Mar 2020		April 2020 to Sept 2020	
**Tumour group**	**Early stage**	**Late stage**	**Early stage**	**Late stage**
Breast	162	71	121	54
Colorectal	53	116	20	57
Lung	98	140	61	114
Prostate	259	139	84	48
**Overall**	**572**	**466**	**286**	**273**

	Oct 2019 to Mar 2020		April 2020 to Sept 2020		Variance (Late stage)
Tumour group	Early stage	Late stage	Early stage	Late stage	
Breast	69.5%	30.5%	69.1%	30.9%	+0.4%
Colorectal	31.4%	68.6%	26.0%	74.0%	+5.4%
Lung	41.2%	58.8%	34.9%	65.1%	+6.3%
Prostate	65.1%	34.9%	63.6%	36.4%	+1.4%
**Overall**	55.1%	44.9%	51.2%	48.8%	+3.9%

**Table 4.** Cancer staging comparison Apr 2020 to Sep 2020 versus Oct 2019 to Mar 2020. Number of cases and percentage breakdown at Stages 1–4.

	Oct 2019 to Mar 2020				Apr 2019 to Sep 2020			
**Tumour group**	**Stage 1**	**Stage 2**	**Stage 3**	**Stage 4**	**Stage 1**	**Stage 2**	**Stage 3**	**Stage 4**
Breast	93	69	46	25	55	66	31	23
Colorectal	21	32	75	41	10	10	37	20
Lung	77	21	51	89	45	16	29	85
Prostate	38	221	84	55	18	66	25	23
**Overall**	**229**	**343**	**256**	**210**	**128**	**158**	**122**	**151**

	Oct 2019 to Mar 2020				Apr 2019 to Sep 2020				Variance (Stage 4)	Greatest shift between stages
Tumour group	Stage 1	Stage 2	Stage 3	Stage 4	Stage 1	Stage 2	Stage 3	Stage 4		
Breast	39.9%	29.6%	19.7%	10.7%	31.4%	37.7%	17.7%	13.1%	+2.4	1–2
Colorectal	12.4%	18.9%	44.4%	24.3%	13.0%	13.0%	48.1%	26.0%	+1.7	2–3
Lung	32.4%	8.8%	21.4%	37.4%	25.7%	9.1%	16.6%	48.6%	+11.2	3–4
Prostate	9.5%	55.5%	21.1%	13.8%	13.6%	50.0%	18.9%	17.4%	+3.6	3–4
**Overall**	**22.1%**	**33.0%**	**24.7%**	**20.2%**	**22.9%**	**28.3%**	**21.8%**	**27.0%**	+6.8	3–4
